# A lifestyle-based prediction model for obesity in Chinese adolescent students 

**DOI:** 10.3389/fspor.2025.1677707

**Published:** 2025-10-30

**Authors:** Mei Jiang, Zhou He

**Affiliations:** ^1^ Sports Institute of Chengdu University of Technology, Chengdu, Sichuan, China; ^2^ School of Mathematics, Southwest Jiaotong University, Chengdu, Sichuan, China; ^3^ Sichuan Province Big Data Research and Joint Application Technology Center of Student Health, Chengdu, Sichuan, China; ^4^ National-Local Joint Engineering Laboratory of System Credibility Automatic Verification, Chengdu, Sichuan, China

**Keywords:** lifestyle behaviors, Chinese students, adolescent students obesity, prediction model, LASSO regression

## Abstract

**Introduction:**

Adolescent obesity has emerged as a critical global public health challenge, necessitating effective tools for early identification and intervention. This study aimed to identify significant contributing factors and develop a predictive model for adolescent obesity using machine learning algorithms.

**Methods:**

An anonymised dataset of 2,338 adolescents was utilised, incorporating variables related to family factors, lifestyle behaviours, and physical fitness scores. Variable selection was performed using LASSO regression with *k*-fold cross-validation, followed by parameter estimation via logistic regression. The optimal classification threshold was determined using the Youden Index.

**Results:**

The final predictors included gender, mother's educational level, parental BMI, weight at age 12, parenting style, weekly sweets consumption frequency, meal duration, sleep duration, and physical fitness score. The model demonstrated robust performance, with an AUC of 0.91, accuracy of 0.86, and sensitivity of 0.84. Subgroup analysis indicated consistent performance across genders, with slightly superior predictive efficacy in males (AUC = 0.912) compared to females (AUC = 0.898).

**Discussion:**

The proposed interpretable framework combines high predictive accuracy and sensitivity, offering a valuable tool for timely identification and intervention in high-risk adolescents. These findings underscore the potential of data-driven approaches in addressing adolescent obesity.

## 1 Introduction

Obesity is a disease that also causes other noncommunicable diseases (1). However, the World Obesity Federation predicts that, based on body mass index (BMI) measurements, more than 750 million children and adolescents aged 5 to 19 worldwide will be overweight or obese by 2035. This equates to two out of every five children globally facing this problem (2).

National Health Commission of the People’s Republic of China has released “The Guidelines for Weight Management (2024 Edition)”, emphasizing the significance of lifestyle behaviour assessment in weight management, including dietary habits, levels of physical activity, quality of sleep, mental health status, and smoking and drinking habits (3). This method enables the early screening of obesity risk in large populations and has the advantages of being convenient and low-cost in comparison to physiological indicator testing.

A Chinese research team developed an obesity prediction model by using baseline and 5-year follow-up data on gender, age, urban/rural residence, and BMI from 88,980 elementary and secondary school students in Yantai City. However, the model achieved modest accuracy (70%), and it only included demographic indicators (4). Meanwhile, a longitudinal data tracking study conducted in Australia examined changes in children’s lifestyle behaviours (dietary, physical activity, and screen time) from ages 2 to 5 years old. Nevertheless, the study’s findings are not applicable to children above the age of 5 years old (5). Despite the prevalence of BMI screening in most states of the United States, the implementation of interventions to enhance it remains limited. Zare et al. examined the predictive ability of BMI in kindergarten children for obesity in those same children in the fourth grade, confirmed the significant role of this indicator, and provided insights for research in Asia (6). While previous studies have explored obesity prediction, their applicability to China’s large population remains limited. Furthermore, the performance of the model, as indicated by factors such as prediction accuracy, can be improved.

From the perspective of lifestyle-related indicators, a meta-analysis demonstrated that higher intake of sugary drinks, fast food, refined grains, and meat was positively associated with obesity, while a higher intake of whole grains and sweet bread was negatively associated with obesity. However, the research conclusions are controversial, with debate surrounding the impact of sweet bread intake on obesity (7). Shorter sleep cycles have been demonstrated to be positively correlated with obesity in preschool and school-age children (8). For children aged 4–12, a sleep duration of less than 10 h is considered to be short; for children aged 13–18, a sleep duration of less than 8 h is short (9). Additionally, a cross-sectional study involving 634 school-aged children aged 6–12 years abroad showed that, after adjusting for confounding factors, family income, moderate physical activity, fast food consumption, and fruit and vegetable intake had a certain impact on the incidence of obesity (10). Wang et al. conducted a cross-sectional investigation to identify potential factors associated with obesity in 9,501 preschool children. Their study found that factors such as eating speed, sleep duration, birthweight, and paternal BMI were associated with overweight and obesity. But the model was found to lack indicators related to physical activity and was not found to be applicable to children or adolescents of other age groups (11). Similarly, a data analysis of school-age children in Jiangsu, China, indicated that daily consumption of sugary drinks and low levels of moderate to vigorous physical activity are positively correlated with obesity (12). Therefore, our study considered multiple indicators, including the frequency of sweet food intake, beverage intake, eating speed and duration, sleep duration, and physical fitness score.

Machine learning presents a promising avenue for advancing obesity risk assessment. Contemporary studies have demonstrated that machine learning algorithms outperform traditional regression models in stratifying obesity risk by integrating multifactorial determinants such as dietary patterns, physical activity levels, and familial influences (13–15). However, many existing approaches face critical limitations. Black-box algorithms prioritize predictive accuracy at the expense of interpretability (13), while biomarker-dependent models remain impractical for large-scale implementation due to cost constraints and contextual adaptability issues (16, 17). Furthermore, few current models account for the distinct metabolic and behavioral phenotypes observed across genders or address the challenges of optimal classification thresholds in imbalanced datasets (18). Thus, the purpose of our study is to propose an obesity risk prediction model for adolescents students from the perspective of lifestyle behaviour assessment, and we hope that this will support schools in implementing relevant health education interventions.

We propose an interpretable prediction framework that combines three methodological innovations to overcome these limitations. First, we use LASSO regression for feature selection to identify the lifestyle behaviors relevant to obesity, including students’ anthropometrics and dietary habits, sleep duration, physical fitness score, and parents’ anthropometrics. Second, Logistic Regression provides a transparent probabilistic classification system with high diagnostic accuracy. Third, we optimize classification thresholds using the Youden index to maximize sensitivity for early risk detection, establishing a dynamic threshold of 0.042. The model demonstrates effective predictive ability for the overall student population, as well as for male and female groups when applied separately. This makes it a useful tool for large-scale school obesity screening.

## 2 Methods

### 2.1 Data description and sources

The research data comes from two parts. The first part consists of questionnaire data collected through on-site surveys during the 2025 Sichuan Province Physical Health Spot-Check and Re-verification Work. The questionnaire data includes demographic characteristics of students aged 12–24 and their parents, such as gender, height, weight, BMI, mother’s education level, and whether the father smokes. The data also includes lifestyle behaviors of students, focusing on diet, sleep, and feeding methods.

The second part of the data consists of physical fitness test scores. This data is sourced from the Sichuan Province Student Physical Health Big Data Center, which is an official, non-public data source. The credibility of the data and research is validated by authoritative entities. The center has anonymized all personal information prior to providing the data, ensuring no information that could directly or indirectly identify individual students is included (including but not limited to names, ethnicity, detailed addresses, or school names). A total of 2,394 items of data, however, 56 items of data were excluded due to inadequate internal consistency or data anomalies, resulting in 2,338 items of data being finally included in this study. This study adopted obesity threshold criteria based on the National Student Physical Health Standard (2014 Revision) (19). Additionally, we analyzed the consistency between these Chinese national standards and the WHO’s growth standards for global adolescent populations (20). The Kappa coefficient was calculated as 0.802, indicating almost perfect agreement between the two classification systems. This demonstrates that the obesity classification criteria adopted in this study are valid and reliable. The dataset was randomly split into two subsets: 70% of the data was used for training, and 30% was reserved for testing. Both training and test sets satisfied the minimum sample size requirements for statistical analysis. To ensure model robustness and mitigate the influence of randomness from a single data partition, we employed k-fold cross-validation (k=10) within the training set during model training.

All statistical analyses and modeling were performed using the R programming language (version 4.2.1). The analysis utilized the following key R packages: glmnet (version 4.1.8) for regularized regression model fitting, and pROC (version 1.18.5), ROCR (version 1.0.11), and reportROC (version 3.6) for model evaluation, ROC curve analysis, and reporting of diagnostic metrics.

### 2.2 Factors in predictive models

This study used the following factor data: demographic information (gender, height, weight, age), lifestyle (parenting style, sleep duration, sweetened drinks frequency per week, fried food frequency per week, sweets frequency per week,physical fitness score, etc), and parents’ demographics data (parents’ height and weight, mother’s educational level, and father’s smoking status). Table 1 lists the variables that are associated with the obesity development. We also use these to develop an obesity prediction model. In this study, we applied the LASSO (Least Absolute Shrinkage and Selection Operator) regression method to identify variables significantly associated with obesity. We will next introduce the LASSO regression method.

**Table 1:** Variables that may be used in the predictive model.

Category		Variable
Child	Demographics	Gender, height, weight, age
	Lifestyle behaviors	Parenting style, sleep duration, sweetened drinks/week, fried food/week, sweets/week, eating speed, meal duration, and eating with distractions, physical fitness score
Parents	Demographics	Height, weight, mother’s educational level, smoking status

### 2.3 LASSO model

LASSO Regression (Least Absolute Shrinkage and Selection Operator) (21), originally proposed by Robert Tibshirani in 1996, represents a regularization technique for linear regression models that has gained widespread application in statistical analysis. The method’s core mechanism involves imposing an $L_{1}$-norm penalty on the regression coefficients, simultaneously achieving coefficient shrinkage and feature selection. This dual functionality enables LASSO to effectively address challenges inherent in high-dimensional datasets (characterized by excessive variables) and data exhibiting multicolsleep hourlinearity. Traditional ordinary least squares regression often encounters significant limitations in such contexts, including multicollinearity effects, difficulties in identifying relevant predictors, and heightened risk of model overfitting. Through its unique regularization approach, LASSO systematically identifies statistically significant features while driving redundant predictor coefficients toward exact zero values, thereby reducing model complexity. This process not only enhances predictive accuracy but also improves model interpretability by producing sparse solutions that explicitly identify key contributing variables.

The objective function of LASSO regression can be formulated as the following optimization problem:

(1) $$\min_{\beta }\left(\frac{1}{2n}\sum_{i=1}^{n}(y_{i}-X_{i}\beta {)}^{2}+\lambda \sum_{\;j=1}^{\;p}|\beta_{j}|\right).$$

In the above Equation 1, $y_{i}$ denotes the dependent variable (response) of the ith sample, and $X_{i}$ represents the corresponding vector of independent variables. The parameter $\beta =(\beta_{1},\beta_{2},\ldots ,\beta_{p}{)}^{T}$ is the vector of regression coefficients to be estimated. The regularization parameter λ≥0 controls the strength of the $L_{1}$ penalty term. Here, n is the total number of samples, and p refers to the dimensionality of the feature space, i.e., the number of independent variables. The objective function of the LASSO regression model comprises two key components: the squared loss function and the $L_{1}$ regularization term. The squared loss function quantifies the discrepancy between the model’s predicted values and the observed response values, serving as a measure of model accuracy. The $L_{1}$ regularization term, on the other hand, introduces a penalty proportional to the sum of the absolute values of the regression coefficients. This penalty encourages sparsity in the estimated coefficient vector by shrinking some coefficients exactly to zero, effectively performing variable selection and yielding a simpler, more interpretable model.

### 2.4 Logistic regression model

Logistic regression is a widely used statistical method for binary classification tasks. Despite its name, it is not a linear regression model but a probabilistic classification algorithm that estimates the probability of an instance belonging to a specific class. The model maps input features to a probability value between 0 and 1 using a logistic (Sigmoid) function, enabling the prediction of discrete outcomes.

The core idea of logistic regression is to model the relationship between input features $X=[x_{1},x_{2},\ldots ,x_{n}]$ (where n is the number of features) and the target variable y∈{0,1}. The model computes a linear combination of the input features and applies the Sigmoid function to transform the result into a probability. The model is defined as follows, as shown in Equations 2, 3:?>

(2) $$z=\beta_{0}+\beta_{1}x_{1}+\beta_{2}x_{2}+\cdots +\beta_{n}x_{n}=\beta^{T}X,$$

(3) $$P(y=1|X;\beta )=\sigma (z)=\frac{1}{1+e^{-z}}.$$

Here, $\beta =[\beta_{0},\beta_{1},\ldots ,\beta_{n}]$ represents the model parameters (including the intercept $\beta_{0}$), and σ(⋅) is the Sigmoid function. The output σ(z) represents the probability that the input X belongs to class 1. A threshold (typically 0.5) is applied to classify the instance: if σ(z)≥0.5, the prediction is class 1; otherwise, it is class 0.

To train the logistic regression model, we minimize a loss function that quantifies the discrepancy between predicted probabilities and true labels. The logarithmic loss (or cross-entropy loss) is commonly used:

(4) $$L(\beta )=-\frac{1}{m}\sum_{i=1}^{m}\left[y^{\left(i\right)}\log({\hat{y}}^{\left(i\right)})+(1-y^{\left(i\right)})\log(1-{\hat{y}}^{\left(i\right)})\right].\;$$

In the Equation 4, *m* is the number of training samples, $y^{\left(i\right)}$ is the true label for the ith sample, and ${\hat{y}}^{\left(i\right)}=\sigma (\beta^{T}X^{\left(i\right)})$ is the predicted probability.

The model parameters β are optimized using gradient descent or its variants (e.g., stochastic gradient descent, Adam). The gradient of the loss function with respect to $\beta_{j}$ is computed as:

$$\frac{\partial L}{\partial \beta_{j}}=\frac{1}{m}\sum_{i=1}^{m}({\hat{y}}^{\left(i\right)}-y^{\left(i\right)})x_{j}^{\left(i\right)}.$$

This gradient is iteratively updated to minimize the loss until convergence.

Logistic regression is particularly suitable for problems where interpretability is critical. For example, in medical diagnosis, the model can quantify the impact of risk factors (e.g., age, blood pressure) on disease probability. This interpretability makes logistic regression a popular choice in domains like healthcare, finance, and social sciences.

### 2.5 Youden index

The Youden Index (Youden’s J statistic) is a widely used metric for evaluating the performance of binary classification models, particularly in scenarios where class imbalance exists or when balancing sensitivity and specificity is critical. It quantifies the ability of a model to correctly distinguish between positive and negative classes by combining sensitivity (true positive rate) and specificity (true negative rate) into a single metric. The Youden Index is defined as:

(5) J=Sensitivity+Specificity−1.

The Equation 5 integrates two critical aspects of diagnostic accuracy into a single scalar value, enabling direct comparison across models or thresholds.

The sensitivity (also known as the true positive rate, TPR) quantifies the model’s ability to correctly identify positive instances:

$$\text{Sensitivity}=\frac{\text{TP}}{\text{TP}+\text{FN}}.$$

The specificity (true negative rate, TNR) measures the model’s capacity to correctly reject negative instances:

$$\text{Specificity}=\frac{\text{TN}}{\text{TN}+\text{FP}}.$$

Here, TP (true positives), TN (true negatives), FP (false positives), and FN (false negatives) are components of the confusion matrix derived from the classification results.

The Youden Index J, ranging from −1 to 1, quantifies the discriminative ability of a binary classification model. A value of J=1 indicates perfect classification, where all samples are correctly predicted (i.e., no false positives or false negatives). Conversely, J=0 corresponds to no discriminative power, equivalent to random guessing, while J<0 suggests performance worse than random, though this is rare in practical applications. This metric is particularly valuable in domains such as medical diagnostics and anomaly detection, where minimizing both false positives (FP) and false negatives (FN) is critical. By explicitly balancing sensitivity and specificity, the Youden Index avoids the pitfalls of accuracy-based metrics, which can be misleading in imbalanced datasets. Its design ensures a robust trade-off between correctly identifying positive cases and avoiding incorrect rejections of negative cases, making it a reliable tool for threshold selection and performance evaluation in real-world scenarios.

Combining logistic regression with the Youden Index primarily aims to optimize the model’s decision threshold, thereby enhancing classification performance. While logistic regression defaults to a threshold of 0.5, this value may not be optimal in practical applications. By integrating the Youden Index, a threshold that maximizes the sum of sensitivity and specificity can be identified, thus improving the model’s classification effectiveness.

The implementation steps are as follows:

• Train the logistic regression model: First, use the training dataset to train the logistic regression model.
• Obtain prediction probabilities: Use the trained model to predict the validation set or test set, and obtain the probability of each sample belonging to the positive class.
• Calculate sensitivity and specificity at different thresholds: Iterate through a series of possible thresholds. For each threshold, classify samples into positive or negative classes based on the predicted probability, and calculate the corresponding sensitivity and specificity.
• Determine the optimal threshold: Using the calculated sensitivity and specificity, identify the threshold that maximizes the Youden Index as the optimal threshold for the model.
• Apply the optimal threshold: Replace the default 0.5 threshold with the optimal threshold found and classify new data.

The advantage of this method is that it considers the balance between positive and negative classes, making it particularly suitable for imbalanced data problems. It can help improve the overall performance and practicality of the model.

## 3 Results

### 3.1 Basic data analysis of variables for predictive models

The variables potentially applicable for developing an obesity prediction model are summarized in Table 1. After data preprocessing, a total of 2,338 samples were retained, with 1,631 (70%) assigned to the training set and 707 (30%) used for internal validation. The descriptive statistics of categorical variables are presented in Table 3, while those of numerical variables are shown in Table 2. Based on the training set, 48.31% of the participants were male and 51.69% were female, an average sleep duration of 533.69 (215.81) min, and a mean physical fitness score of 76.12 (SD 14.36). The variable design of the study includes more lifestyle-related indicators for adolescent students than previous studies. The dataset was partitioned appropriately, and the training and test sets demonstrated good consistency, providing a solid foundation for the development of obesity prediction models.

**Table 2:** Numerical variables that can be used in predictive modeling.

Category	Variable	Training set (N=1,631)	Test set (N=707)
		Mean (SD)	Mean (SD)
Child	Weight at age 12	45.08 (9.56)	44.80 (9.38)
	Sleep duration	533.69 (215.81)	528.09 (206.01)
	Physical fitness score	76.12 (14.36)	76.69 (13.88)
Parents	Father’s BMI	24.80 (9.77)	24.02 (6.19)
	Mother’s BMI	22.73 (4.98)	22.49 (4.09)

**Table 3:** Categorical variables that can be used in predictive modeling.

Category	Variable	Characteristics	Training set (N=1,631)	Test set (N=707)
			N (%)	N (%)
Child	Gender	Male	788 (48.31)	333 (47.10)
		Female	843 (51.69)	374 (52.90)
	Parenting style	Parents	1,130 (69.28)	487 (68.88)
		Grandparents	489 (29.98)	217 (30.69)
		Childcare	12 (0.74)	3 (0.43)
	Sweetened Drinks/Week	Never drink	321 (19.68)	131 (18.53)
		Less than once a day	1,195 (73.27)	524 (74.12)
		More than once a day	115 (7.05)	52 (7.35)
	Fried Food/Week	Never drink	391 (23.97)	144 (20.37)
		Less than once a day	1,165 (71.43)	523 (73.97)
		More than once a day	75 (4.60)	40 (5.66)
	Sweets/Week	Never drink	250 (15.33)	92 (13.02)
		Less than once a day	1,025 (62.84)	449 (63.51)
		Once a day	284 (17.41)	142 (20.08)
		More than once a day	72 (4.41)	24 (3.39)
	Eating speed	Very slow	31 (1.90)	11 (1.56)
		Slow	136 (8.34)	56 (7.92)
		Moderate	1,003 (61.50)	444 (62.80)
		Fast	390 (23.91)	169 (23.90)
		Very fast	71 (4.35)	27 (3.82)
	Meal duration	Less than 10 min	310 (19.01)	130 (18.39)
		10–20 min	1,034 (63.40)	462 (65.34)
		20–30 min	263 (16.13)	109 (15.42)
		More than 30 min	24 (1.47)	6 (0.85)
	Eating with Distractions	Yes	878 (53.83)	371 (52.48)
		No	753 (46.17)	336 (47.52)
Parents	Mother’s education	Junior high school or below	888 (54.45)	357 (50.50)
		High school	526 (32.25)	264 (37.34)
		Junior college	115 (7.05)	57 (8.06)
		University and above	102 (6.25)	29 (4.10)
	Smoking status	Yes	1,018 (62.42)	434 (61.39)
		No	613 (37.58)	273 (38.61)

### 3.2 Variable selection

**Figure 1:**
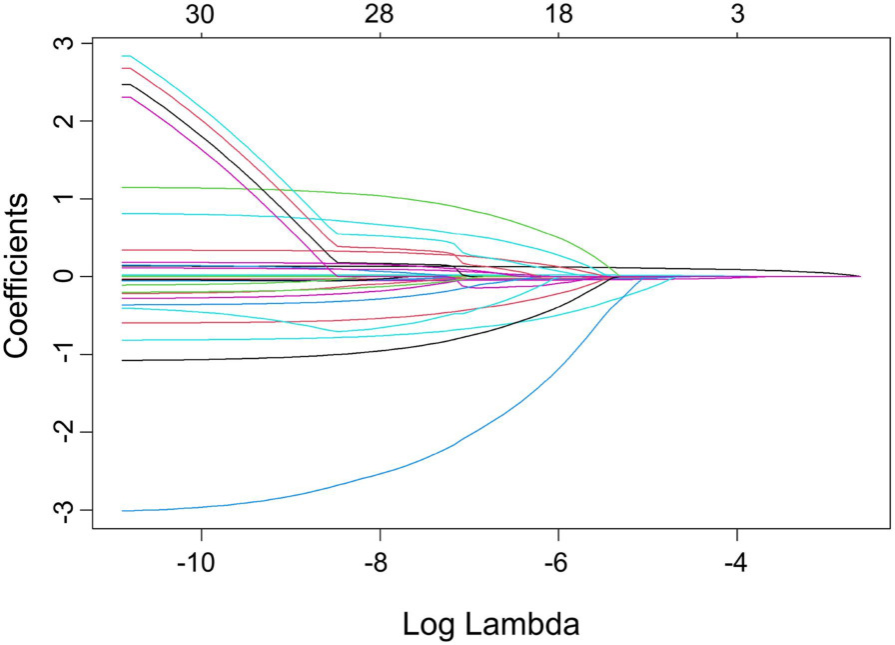
Variable selection process for the obesity prediction model.

**Figure 2:**
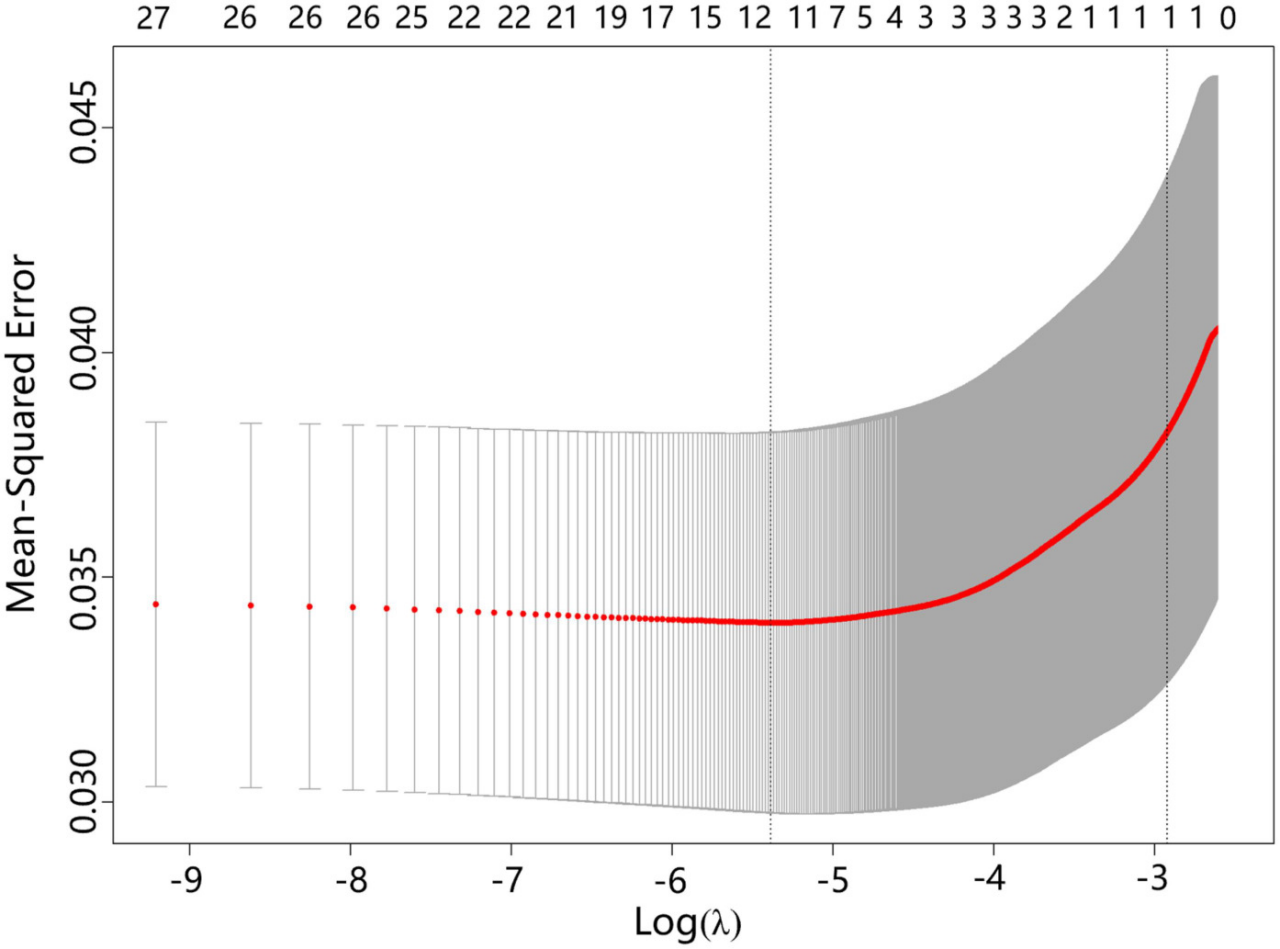
Regularization path of parameters in the obesity prediction model.

The LASSO model included ten variables to screen for those significantly associated with obesity. These variables covered students’ demographic information, dietary habits, sleep conditions, physical conditions, and their parents’ demographics, mothers’ education levels, and fathers’ smoking statuses. Categorical variables were handled using dummy variable encoding. Figure 1 illustrates the variable shrinkage process in the LASSO model, and Figure 2 shows the corresponding regularization path. The estimated value of λ is $4.58\times 10^{-3}$, and the variable selection results are shown in Table 4. The results indicate that students’ gender, weight at age 12, parenting style, sleep duration, frequency of sweets per week, meal duration, and physical fitness score, as well as parents’ BMI and mothers’ educational attainment, are significantly associated with students’ obesity. This highlights the importance of cultivating healthy lifestyle behaviors, including proper diet, exercise, physical activity and sleep. The predictive model in this study will be constructed based on these indicators.

**Table 4:** Variables significantly associated with obesity selected by the LASSO method.

Category		Variable
Child	Demographics	Gender, weight at age 12
	Lifestyle behaviors	Parenting style, sleep duration, sweets/week, meal duration, physical fitness score
Parents	Demographics	BMI, mother’s education level

### 3.3 Prediction model results

**Figure 3:**
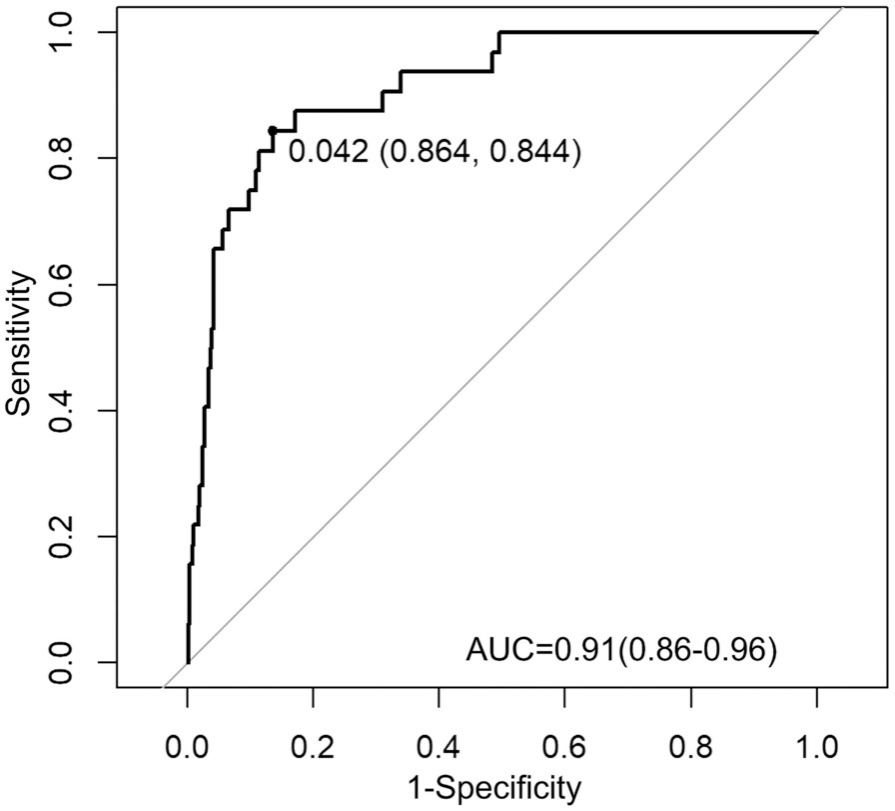
ROC curve of the obesity prediction model performance.

For the variables selected using the LASSO regression method, we established a binary classification model using logistic regression combined with the Youden Index. The classification threshold calculated by the Youden Index was 0.042. The model achieved an accuracy of 0.86, a sensitivity of 0.84, and a specificity of 0.86. The ROC curve of the prediction model is shown in Figure 3, and the AUC value was 0.91, illustrating the strong discriminative ability. Table 5 shows the threshold selection process for the obesity prediction model. Table 6 shows the parameters of the obesity prediction model after training on the training set, from which it can be seen that the higher the physical fitness score and the lower the parents’ BMI, the lower the likelihood of adolescent obesity. Longer sleep duration is associated with a higher probability of obesity, which can be attributed to the fact that the sleep duration range observed in this study primarily falls within 7–11 h. Table 2 also indicates that the average sleep duration among adolescents is approximately 9 h. This finding is consistent with the U-shaped relationship between sleep duration and obesity reported in the Ref. (22), as the observed positive correlation corresponds to the right segment of this U-shaped curve.

**Table 5:** Performance of the obesity prediction model at different thresholds.

Threshold	0.01	0.03	0.05	0.07	0.09	0.11	0.13	0.15	0.17	0.19
Accuracy	0.528	0.813	0.874	0.907	0.934	0.943	0.941	0.941	0.946	0.948
Sensitivity	0.969	0.875	0.812	0.719	0.656	0.656	0.500	0.406	0.406	0.344
Specificity	0.507	0.810	0.877	0.916	0.947	0.957	0.961	0.966	0.972	0.976
PPV*	0.085	0.179	0.239	0.287	0.368	0.420	0.381	0.361	0.406	0.407
NPV*	0.997	0.993	0.990	0.986	0.983	0.983	0.976	0.972	0.972	0.969

* PPV, positive predictive value; NPV, negative predictive value.

**Table 6:** Parameters of the optimal logistic regression model for obesity prediction.

Variable	Characteristics	Estimates	Standard error	95% CI	p-value
Gender	Male (reference)			
	Female	0.566	0.262	[0.052, 1.080]	0.031
Mother’s education	Junior high school or below (reference)				
	High school	−1.002	0.385	[−1.800, −0.28]	0.009
	Junior college	−0.141	0.580	[−1.200, 1.103]	0.807
	University and above	−0.977	0.745	[−2.640, 0.337]	0.189
Parenting style	Parents (reference)				
	Grandparents	0.279	0.327	[−0.376, 0.912]	0.394
	Childcare	1.204	0.994	[−1.029, 2.982]	0.225
Weight at age 12		0.133	0.013	[0.110, 0.160]	<0.01
Father BMI		0.014	0.013	[−0.024, 0.033]	0.284
Mother BMI		0.061	0.021	[0.020, 0.103]	0.003
Sweets/week	Never drink (reference)				
	Less than once a day	−0.137	0.407	[−0.909, 0.697]	0.736
	Once a day	−0.527	0.578	[−1.708, 0.583]	0.361
	More than once a day	1.283	0.627	[0.053, 2.513]	0.041
Meal duration	Less than 10 min (reference)				
	10 to 20 min	−0.291	0.364	[−0.990, 0.446]	0.423
	20 to 30 min	−0.431	0.558	[−1.578, 0.632]	0.440
	More than 30 min	−0.121	0.141	[−0.398, 0.156]	0.391
Sleep duration		4.08$\times 10^{-4}$	6.77$\times 10^{-4}$	[−0.0010, 0.0017]	0.547
Physical fitness score		−0.047	0.009	[−0.065, −0.029]	<0.01

To evaluate potential gender-based performance variations, we conducted stratified analyses. In the female subgroup (N=374), the model maintained high predictive accuracy (Accuracy = 0.896, AUC = 0.898), with sensitivity of 0.786 and specificity of 0.900. The male subgroup (N=333) showed comparable performance (Accuracy = 0.826, AUC = 0.912), with sensitivity of 0.889 and specificity of 0.822. The detailed performance results of the obesity prediction model for sex-stratified and overall data are presented in Table 7.

**Table 7:** Gender-stratified model performance metrics.

Group	Size	Accuracy	Sensitivity	Specificity	PPV*	NPV*	AUC*
Overall	707	0.863	0.844	0.864	0.227	0.991	0.911
Male	333	0.826	0.889	0.822	0.221	0.992	0.912
Female	374	0.896	0.786	0.900	0.233	0.991	0.898

* PPV, positive predictive value; NPV, negative predictive value; AUC area under the curve.

**Figure 4:**
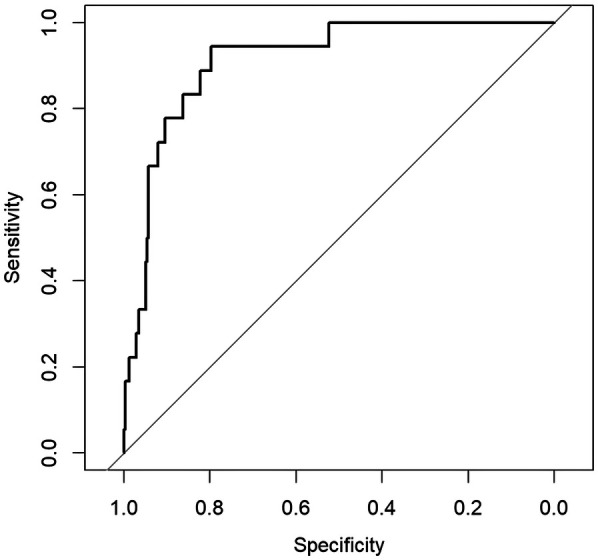
ROC curve for the obesity prediction model in males.

**Figure 5:**
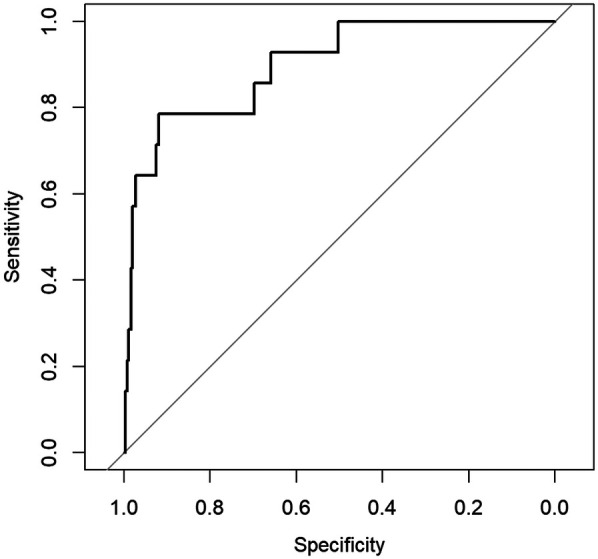
ROC curve for the obesity prediction model in females.

**Figure 6:**
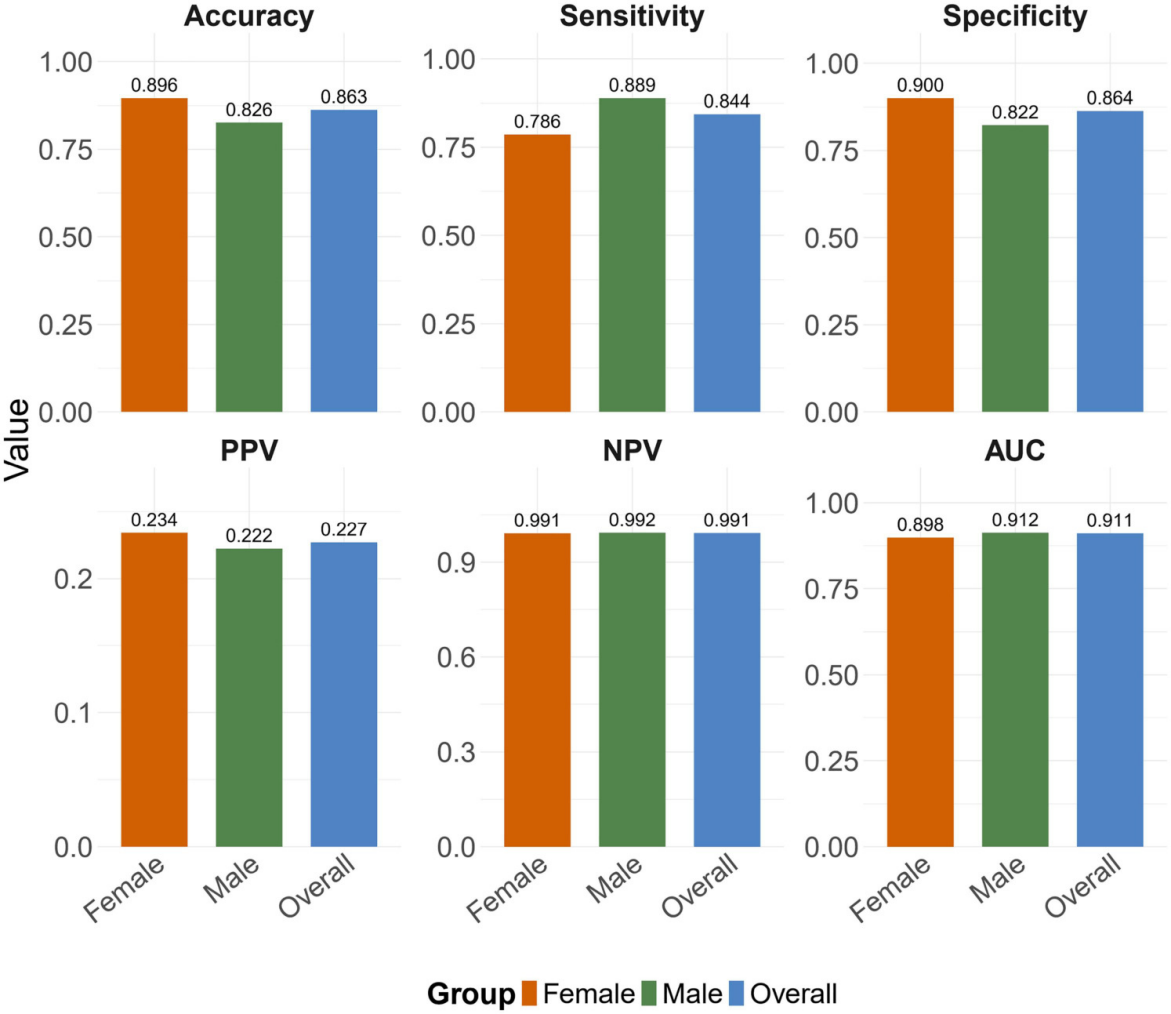
Summary of performance metrics of the obesity prediction model across different datasets. Abbreviations: PPV, positive predictive value; NPV, negative predictive value; AUC, area under the curve.

The ROC curves illustrating the sex-specific predictive performance of the obesity prediction model in males and females are presented in Figures 4 and 5, respectively. Notably, the model demonstrated consistent predictive power across genders, as evidenced by the ROC curves and the stable performance metrics in Table 7. The highest AUC value (0.912) was observed in the male group, with a female AUC of 0.898. These stratified results confirm the model’s reliability across demographic subgroups. Figure 6 presents a multifaceted comparison of performance metrics, visually synthesizing these findings. The visualization highlights the model’s consistent accuracy across diverse demographic groups, demonstrating stable discriminative capability irrespective of gender.

These comprehensive analyses collectively indicate that the obesity prediction model delivers reliable performance for both male and female adolescents. The observed gender-based variations in specific metrics may reflect biological differences in obesity manifestation rather than model limitations, a hypothesis that warrants investigation in future physiological studies. The consistent AUC values above 0.85 across all groups satisfy conventional criteria for ’good’ to ’excellent’ discriminatory power (23).

## 4 Discussion

This study presents an obesity prediction model containing more lifestyle behavior indicators for Chinese adolescent students through an LASSO-logistic regression framework optimized by the Youden Index. The model shows predictive ability (AUC = 0.911), with gender-specific performance difference (females: AUC = 0.898; males: AUC = 0.912). The optimized classification threshold (0.042) prioritizes sensitivity (0.844), emphasizing early detection ability for high-risk individuals. The model’s superior AUC outperforms traditional BMI-based approaches (24, 25) and rivals advanced machine learning frameworks (26, 27). This balance of accuracy and interpretability addresses a key limitation in complex models, which often sacrifice applicability to adolescent student populations for predictive gains. The low classification threshold prioritizes sensitivity, ensuring early identification of at-risk adolescents during critical developmental windows. Meanwhile, the standard errors of the predictive model are comparatively small, indicating relatively high stability. However, the confidence intervals of some variables may still include zero. This may reflect more complex relationships between these variables and the outcome variable in the dataset, or could be due to imbalanced distribution of variable values (e.g., some values being relatively rare) or subjectivity in questionnaire responses. Therefore, this issue cannot be solely attributed to the modeling algorithm.

The model established in this study demonstrated robust predictive performance within the Chinese adolescent population. However, its generalizability to other populations requires further consideration. Compared with Western populations, Chinese adolescents exhibit differences in obesity prevalence, genetic background, and lifestyle. Studies (13, 14) that presented obesity prediction models and related influencing factors in Mexican populations revealed that certain risk factors demonstrate cross cultural consistency, such as high calorie diets and low levels of physical activity. This indicates that the core mechanism of obesity-an imbalance between energy intake and expenditure-is universal. This model also accounted for parental obesity, which may be related to genetic factors. Nevertheless, population specific factors were also identified. We incorporated factors such as sleep duration and maternal education level, reflecting that adolescents may be more susceptible to influences from rapid economic development and cultural habits. In contrast, Dirik’s model (14) incorporated behavioral factors such as alcohol consumption and daily electronic device usage. These discrepancies may stem from differences in the age of the study populations, cultural habits, and social environments. Overall, the core lifestyle factors included in our model demonstrate sound rationale. However, calibration and adjustment, such as incorporating population specific social environmental and behavioral variables, are necessary when applying the model across different cultures or regions. Future research should validate this model framework in broader international cohorts and develop dynamic prediction tools adaptable to diverse population characteristics.

The importance of weight at age 12 as a predictor is supported by longitudinal evidence showing that adolescents obesity trajectories strongly correlate with adult obesity risk (25, 28). This highlights the importance of monitoring health in early life. The influence of maternal weight is consistent with the well-documented familial obesity transmission mechanisms (29, 30), in which shared dietary patterns, physical activity habits, and genetic predispositions play pivotal roles. Conversely, the protective effect of maternal education level reflects socioeconomic buffers against obesity-promoting environments (31), as higher education is associated with greater health literacy and resource allocation. Behavioral factors, such as fast eating speed and low physical fitness, are supported by mechanistic studies that link these behaviors to energy imbalance and metabolic dysregulation (32, 33). Fast eating may interrupt satiety signaling, and insufficient physical activity contributes to energy imbalance, both of which are crucial drivers of adiposity.

To further evaluate the predictive ability and generalizability of the obesity prediction model, this study assessed the model’s independent performance in male and female populations. As shown in Figure 6, the model’s predictive accuracy was slightly better in the female population than in the male population (Accuracy = 0.896 versus 0.826). These differences align with biological susceptibility to obesity (34, 35) and behavioral heterogeneity (36), such as higher exercise intensity and greater energy expenditure variability in males, whereas females typically exhibit more consistent dietary restraint and sleep-related metabolic stability, which may make the model more generalizable in the female population. Additionally, biological heterogeneity in fat distribution may also impact model accuracy. These findings suggest that gender-specific characteristics may play a role in developing intervention strategies.

Although this study has certain advantages, it also has limitations. Due to the influence of traditional culture, lifestyle behaviors in China may exhibit unique patterns. Therefore, while the model shows promise for broader application within China, its performance may be limited in other countries or populations [such as South African adolescents (37) or Turkish cohorts (38)]. That said, the overall modeling approach is generalizable. Future studies may incorporate variables specific to Western populations to improve cross-cultural applicability. This paper does not discuss in depth the physiological mechanisms behind the differences in prediction accuracy between males and females, nor does it address age-specific issues. In the future, combining this framework with explainable machine learning (27) or deep learning (26) could better address nonlinear interactions. Additionally, by distinguishing physiological characteristics, further refining lifestyle-related indicators tailored to different genders and educational levels (middle school, high school) could lead to the development of more precise predictive models. The accuracy and granularity of categorical variables represent a potential limitation. The use of predefined categories, although necessary for analysis, may reduce statistical power and obscure more complex, non-linear relationships between the variables and the outcome. Future studies should further refine the design of categorical variables to improve the stability and predictive accuracy of the model.

Our model incorporates multidimensional lifestyle indicators to construct an obesity prediction model, which has the advantages of high accuracy and low threshold in all adolescents and in different gender groups. This highlights its application value in large student populations.

## 5 Conclusion

In summary, this study has examined the influence of key factors such as family environment, dietary habits, sleep duration, and physical fitness score on adolescent obesity. Furthermore, it demonstrates that combining LASSO regression for variable selection, logistic regression for probabilistic modeling, and the Youden Index for threshold optimization yields a highly effective tool for predicting childhood obesity. The model demonstrates a strong discriminative ability (AUC = 0.911), coupled with balanced sensitivity and specificity. These characteristics contribute to its potential as a considerable asset for the identification of early risks and appropriate interventions in large-scale adolescent student populations.

## Data Availability

The datasets presented in this article are not readily available because Our data comes from the Sichuan Province Student Physical Health Big Data Center, which is an institution under the Sichuan Provincial Department of Education. This data is not publicly available, so we apologize for not being able to disclose it. Requests to access the datasets should be directed to zhou.he@swjtu.edu.cn.
